# A Minimal Parameter Set Facilitating Early Decision-making in the Diagnosis of Hemophagocytic Lymphohistiocytosis

**DOI:** 10.1007/s10875-021-01005-7

**Published:** 2021-03-29

**Authors:** Bas M. Smits, Joris van Montfrans, Samuel A. Merrill, Lisette van de Corput, Mariëlle van Gijn, Andrica de Vries, Cor van den Bos, Floor Abbink, Renate G. van der Molen, Natasja Dors, Caroline Lindemans, Jaap J. Boelens, Stefan Nierkens

**Affiliations:** 1 Center of Translational Immunology, University Medical Center Utrecht, Heidelberglaan 100, 3584CX Utrecht, The Netherlands; 2 Department of Pediatric Immunology and Infectious Diseases, UMC Utrecht, Utrecht, The Netherlands; 3 Division of Hematology/Oncology, West Virginia University, Morgantown, WV USA; 4 Department of Medical Genetics, University Medical Center Utrecht, Utrecht, The Netherlands; 5 Department of Pediatric Oncology, Erasmus University Medical Center, Rotterdam, The Netherlands; 6 Princess Máxima Center for Pediatric Oncology, Utrecht, The Netherlands; 7 Department of Pediatric Oncology, Amsterdam University Medical Center, Amsterdam, The Netherlands; 8 Department of Laboratory Medicine, Laboratory for Medical Immunology, Radboud Institute for Molecular Life Sciences, Radboud University Medical Center, Nijmegen, The Netherlands; 9 Department of Pediatric Oncology, Radboud University Medical Center, Nijmegen, The Netherlands; 10 Stem Cell Transplantation and Cellular Therapies, Memorial Sloan Kettering Cancer Center, New York, NY USA

**Keywords:** HLH, hemophagocytic lymphohistiocytosis, diagnostic criteria, clustering analysis

## Abstract

Hemophagocytic lymphohistiocytosis (HLH) is a life-threatening immune dysregulation syndrome characterized by uncontrolled immune cell activation. Timely diagnosis is important, since early treatment can improve survival rates. However, completing all assessments needed to reach ≥5 positive criteria out of the 8 HLH-2004 criteria can be time consuming and may delay timely initiation of treatment. Hence, we applied a data-driven approach to identify a minimal parameter set for early decision-making towards the initiation of HLH-specific treatment. We retrospectively evaluated 165 patients from five Dutch tertiary hospitals with suspected HLH. Sixteen pHLH (median age 0.5 years) and 70 sHLH patients (median age 8.7 years) were identified using the HLH-2004 criteria. Clustering analysis and multi-receiver operator characteristics were used to identify parameters distinctive of HLH. The presence of either increased ferritin, cytopenia in ≥2 lineages, or splenomegaly distinguished HLH from non-HLH cases with a negative predictive value of 100%. A minimal parameter set consisting of 2 major criteria (phagocytosis and splenomegaly) and 3 minor criteria (cytopenia, increased ferritin, and increased triglycerides/low fibrinogen) predicted HLH with 95% (88–99) sensitivity and 94% (86–98) specificity. This finding was replicated in an independent retrospective validation cohort of 109 US patients (*n* = 109). By dividing a subset of the HLH-2004 criteria into major and minor criteria, this strategy uses the evaluation of less than 5 criteria to quickly identify patients with HLH. When confirmed in a prospective setting, this approach could be of value for timely diagnosis and treatment of HLH.

**Supplementary Information:**

The online version contains supplementary material available at 10.1007/s10875-021-01005-7.

## Introduction

Hemophagocytic lymphohistiocytosis (HLH) is a life-threatening immune dysregulation syndrome characterized by uncontrolled immune cell activation. [1, 2] Activated lymphocytes and macrophages produce a cytokine storm that induces hemophagocytosis and tissue phagocytosis by overactivated macrophages. [1, 3, 4] HLH eventually results in multiple organ failure that culminates in mortality in up to 50% of affected children and adults. [1, 3, 5, 6]

The two subtypes of HLH include primary HLH (pHLH) and secondary HLH (sHLH). pHLH is a group of diseases caused by germ line mutations in genes involved in vital immunologic pathways. [7, 8] Several pHLH disorders are clustered as familial HLH, in which defective T and NK lymphocyte function causes a failure in the termination of the immune response as well as persistent antigenemia that stimulates the immune system and induces the cytokine storm. [9–11] Secondary HLH can be induced by infection (42%), cancer (40%), or autoimmune disease (11%). [1, 4, 9–12] [4, 13]

The diagnosis of HLH forms a challenge since many of its symptoms are nonspecific. [14, 15] HLH is currently diagnosed according to the presence of at least 5/8 of the HLH-2004 criteria: fever, splenomegaly, increased ferritin, high soluble cluster of differentiation 25 (sCD25), cytopenia in at least two cell lineages, tissue phagocytosis, low NK lymphocyte–mediated lysis of target cells, and either high triglycerides or low fibrinogen. [16] Since the triggers for HLH vary, it is assumed to be a heterogeneous disease and there is debate whether this heterogeneity has implications for the diagnostic process. [17, 18] For example, there is evidence that patients with malignancy-induced HLH present with different HLH-related parameters when compared to HLH patients with other underlying diseases. [18–20] Lastly, the diagnostic properties of the NK lysis assays and the sCD25 assays remain uncertain for the diagnosis of HLH. These laboratory tests can be time consuming and are restricted to specialized centers, which may delay definitive diagnosis and treatment. [18–21]

Early recognition of HLH is imperative as timely administration of immunosuppressant drugs prevents aggravation of immune dysregulation. This was suggested in a previous retrospective analysis showing a correlation between early etoposide administration and survival. [12]

Since the current HLH criteria involve a subset of laborious tests, and early treatment is essential, the question arises whether a subset of the currently used HLH criteria can be used as a reliable first screening to assess the risk for HLH.

Hence, the objective of this study was to identify a minimal parameter set required to predict HLH that could serve as a tool for early therapeutic decision-making. To this end, a data-driven statistical analysis approach was applied in a Dutch retrospective discovery cohort of HLH-suspected children and adults and the determined minimal parameter set was subsequently confirmed in an American retrospective cohort of HLH suspected adults.

## Methods

### Clinical Record Review and Cohort Acquisition

After approval of the medical ethical committee (METC nr. 17/111), we retrospectively evaluated the clinical records of 264 patients from 5 academic medical centers in the Netherlands for whom NK/lymphocyte function tests or sCD25 assays had been performed between 2006 and 2016. After reviewing the patient records, the records of 99 patients were excluded since they were either not evaluated according to the HLH-2004 criteria or too many HLH-2004 criteria were missing to exclude HLH. The records of 165 patients were selected for further review. Fulfilment of ≥5 of the 8 HLH-2004 criteria and/or confirmed germline mutations associated with pHLH were used to establish HLH diagnosis. The results of all 8 HLH-2004 criteria assessments were collected from the electronic records together with patient characteristics such as age, gender, and final diagnosis.

### NK/Lymphocyte Function and sCD25 Analyses

NK lymphocyte functional testing was performed in a central diagnostic reference center (Laboratory of Translational Immunology, UMC Utrecht) using CD107a degranulation and NK target cell lysis assays. For both assays, PBMC’s were isolated and cell subsets were counted. For the CD107a assay, PBMC’s were incubated with K562 tumor cells and CD8+ CD107a+ cells were counted using flow cytometry. For the NK lysis assay, two effector (NK lymphocytes) vs target (K562 tumor cells) ratios (1:1 and 2:1) were used and NK lysis was analyzed using Celltrace violet© and flow cytometry. Moreover, NK lymphocyte function panels from healthy siblings that were not investigated for HLH were included for the analysis of the sensitivity and specificity of the NK lymphocyte function assays.

sCD25 was measured in individual hospitals using the Diaclone© (upper limit of normal: 2500 pg/mL) or Immulite© (upper limit of normal: 7500 pg/mL) enzyme-linked immunosorbent assays (ELISA) according to the manufacturer’s instructions. We normalized the data by calculating a fold change relative to the upper limit of normal of the used test kit.

### Validation Cohort

The validation cohort consisted of 109 sHLH patients and 38 non-HLH patients from the Hematology Division of Johns Hopkins Hospital in Baltimore, MD. These patients were retrospectively acquired from billing and lab results and classified according to the HLH-2004 criteria between January 1, 2009, and August 1, 2018, as previously published. [22] Diagnostic criteria were recorded and peak values for ferritin and triglycerides and fibrinogen nadir values were also noted. HLH was defined by the HLH-2004 criteria. Data collection was approved by the Johns Hopkins Institutional Review Board. Data on the patients was de-identified by the treating physician before data analysis.

### Statistics

Statistics were performed in R studio 1.1.456, which was used to compare proportions of positive parameters between the different groups of HLH patients and produce a heatmap of these parameters. Then, the “mixomics” package was used for a partial least squares discriminant analysis (PLS-DA) to find characterizing parameters in a supervised method. Furthermore, the “psych” and “GPArotations” packages were used to perform a principal component analysis (PCA), after single stochastic regression imputation of missing data with the “amelia” package, to find characterizing parameters in an unsupervised method. The first three principal components were used in further analyses since they explained most of the variance within the dataset.

Multi-receiver operator characteristics (multiROC) curves were calculated by using generalized linear modelling with pROC and R’s inbuilt statistics packages to compare the diagnostic models. Lastly, cutoff values for NK/lymphocyte function were defined by calculating the ∆ and the fold change of the 1:1 vs 2:1 dilution. R’s “Optimal.Cutpoints” and “epiR” packages were used to calculate sensitivity and specificity of the proposed new algorithms.

## Results

### Prevalence of the 8 HLH-2004 Criteria in HLH Subgroups

One hundred sixty-five HLH suspected patients were included in the primary cohort. After evaluating the HLH-2004 criteria, we had 79 non-HLH and 86 HLH patients (16 pHLH and 70 sHLH). The median age of the pHLH group was 0.5 (0–50.7) years, 8.7 (0–83) years in the sHLH group and 9.7 (0–84) years in the non-HLH group. The type of pHLH and the affected gene and genetic variants of the pHLH patients included in this study are reported in supplementary table 1 and included pathologic mutations in PRF1, UNC13D, STX11, and STXBP2. The most common primary diagnosis in the non-HLH group was either autoimmune disease (20.8%) or immunodeficiency (20.8%), whereas infection (27%) and malignancy (24.3%) were the most common causes of sHLH (Table 1). In 62% of the HLH cases (ranging from 58.8 to 100% among subgroups), hemophagocytosis was observed, either in bone marrow aspirates and/or other affected tissue. Patients with sHLH induced by autoimmune disease fulfilled the cytopenia criterion less often and patients with sHLH induced by infections more often had normal NK lysis assays, suggesting that there was significant variation in positive criteria between HLH subgroups. Moreover, significant variation also existed within the HLH subgroups, denoted by the large standard deviations that were found. This was underlined by the fact that the MHscore, a diagnostic tool that differentiates pHLH from MAS, could not differentiate between pHLH and sHLH in this cohort. [23] The score showed an AUC of 0.709, pHLH patients had a median score of 78.5 (60–99.25), and sHLH patients had a median score of 60 (34–72). To identify common denominators of HLH between these groups, we first tried a steered approach, followed by several clustering methods.

**Table 1:** Summary statistics on included patients. Non-HLH patients were defined as patients who did not meet the HLH-2004 criteria. The type of underlying disease was defined as the primary diagnosis that the patient was suffering from (e.g., a patient suffering from SLE and infection was scored as autoimmune; AI) The largest variation in symptom positivity was seen in the presence of cytopenias and an aberrant NK lysis assay. Moreover, splenomegaly, cytopenia, and elevated ferritin were frequently encountered in HLH patients

	Non-HLH (*n* = 79)	pHLH (*n* = 16)	AI (*n* = 11)	Infection (*n* = 20)	PID (*n* = 7)	Cancer (*n* = 18)	Unknown (*n* = 14)
Median age (range)	9 (0–84)	1 (0–51)	16 (1–40)	7 (0–47)	4 (0–64)	15 (1–83)	2 (0–58)
Gender (male)	64.5%	50%	63.6%	50%	100%	61.1%	71.4%
Fever > 38.5 °C (NA)	62.0% (0)	93.8% (0)	100% (0)	95.0% (0)	71.4% (0)	94.4% (0)	76.9% (1)
Splenomegaly (NA)	20.3% (0)	81.3% (0)	72.7% (0)	80.0% (0)	85.7% (0)	77.8% (0)	78.6% (0)
Proven HLH on biopsy (NA)	5.3% (41)	64.3% (2)	66.7% (2)	58.8% (0)	100% (0)	60.0% (3)	71.4% (0)
Cytopenias ≥ 2 (NA)	20.3% (0)	93.8% (0)	45.5% (0)	68.4% (1)	100% (0)	83.3% (0)	64.3% (0)
Ferritin ≥ 500 (NA)	67.7% (14)	100% (0)	90.9%(0)	100% (0)	50% (1)	100% (0)	100% (0)
Ferritin ≥ 1000 (NA)	43.1% (14)	81.3% (0)	81.8%(0)	80% (0)	50% (1)	83.3% (0)	85.7% (0)
- Mean ferritin levels (SD)	5020 (13662)	3379 (2597)	12,106 (10476)	28,689 (101621)	5576 (9017)	7510 (7825)	11,139 (21214)
⇑ Triglycerides/⇓ fibrinogen (NA)	31.8% (35)	86.7% (2)	63.6% (0)	68.4% (1)	66.7% (1)	64.7% (1)	54.5% (3)
- Mean triglyceride levels (SD)	2.47 (1.90)	4.65 (2.11)	4.05(2.23)	4.14(1.62)	2.93(3.03)	4.28(2.53)	3.70 (1.90)
- Mean fibrinogen levels (SD)	3.97 (2.54)	2.81 (3.46)	2.51(1.58)	2.63(1.61)	1.90(1.45)	3.39(1.60)	5.25 (5.98)
⇑ sCD25 (NA)	50.0% (21)	100% (3)	66.7% (2)	87.5% (4)	83.3% (1)	100% (3)	83.3% (2)
- Mean X upper reference value (SD)	4.9 (7)	24.0 (22)	6.2 (5)	13.7 (11)	23.0 (34)	15.0 (13)	18.5 (22)
Aberrant NK lysis assay (NA)	19.4% (48)	75% (12)	37.5% (3)	0% (9)	40% (2)	60% (13)	71.4% (6)
Median # of positive criteria (range)	2 (0–4)	7 (3–7)	5 (5–7)	6 (5–7)	5 (5–7)	6 (5–7)	5 (5–7)
CNS involvement (NA)	0% (49)	60% (1)	25% (3)	45.5% (9)	0% (2)	50% (6)	41.7% (1)

The steered approach consisted of an evaluation of parameters that are readily available in the clinical setting: elevated ferritin, cytopenias in ≥2 cell lines, and splenomegaly. We hypothesized that these criteria may guide decision-making towards additional HLH diagnostics. First, we calculated the diagnostic properties of ferritin as sole marker for HLH. We set the sensitivity to 90% and found that a ferritin value 787 μg/L enabled us to identify patients with HLH with 48% specificity and an AUC of 0.693. To improve this, we then added the other criteria and observed that by adding splenomegaly or severe cytopenia in >2 lineages (according to the HLH-2004 criteria) to the model, the cutoff for ferritin could be increased to 1000 μg/L. The presence of either splenomegaly, severe cytopenia in >2 lineages (according to the HLH-2004 criteria), or increased ferritin (>1000 μg/L) yielded 100% sensitivity and 65% specificity with a negative predictive value of 100% for HLH in the discovery cohort (Supplementary, table 2).

### Hierarchical Clustering

**Fig. 1:**
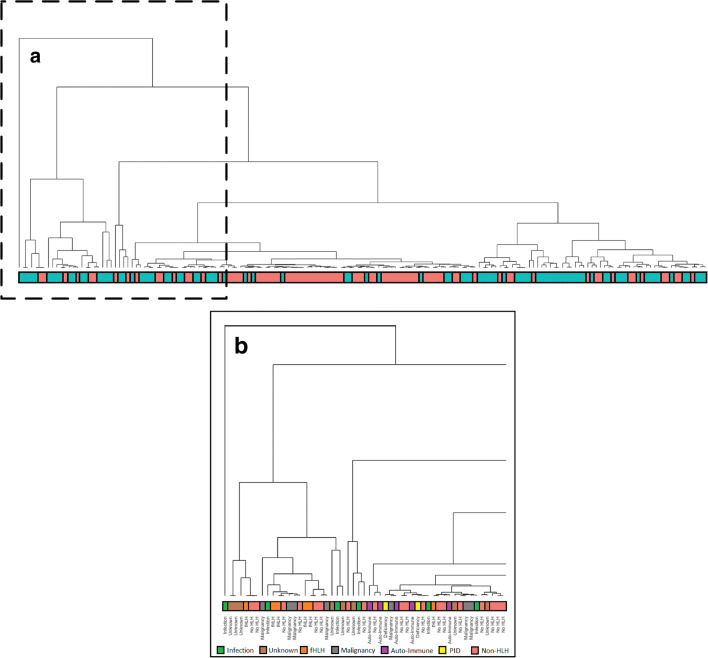
Hierarchical clustering dendogram that could moderately separate non-HLH patients (red) from HLH patients (blue). This clustering strategy could not distinguish pHLH from sHLH patients (A) nor the subtypes of sHLH (B), based on these parameters

Different clustering analysis methods were used to determine the parameters that could distinguish between HLH and non-HLH patients and the different subtypes of HLH. First, hierarchical clustering was applied to the 8 HLH-2004 criteria, CNS symptoms, NK lymphocyte numbers, and bilirubin levels. We found that even though no specific clusters were formed, hierarchical clustering was moderately capable at separating the non-HLH patients from the HLH patients (Fig. 1A). However, hierarchical clustering did not separate the pHLH from sHLH patients (Fig. 1B), nor other subgroups within the HLH spectrum. This suggests that there is no specific set of parameters that can distinguish the separate forms of HLH within the HLH-2004 criteria.

### Supervised Clustering with Dimension Reduction

Secondly, PLS-DA was used to maximize the chance of finding discriminating clusters of criteria that could define the sHLH subgroups and distinguish between sHLH and pHLH, but none could be found (Supplementary, fig. 1). The criteria that could identify HLH patients in general overlapped excessively for the pHLH and sHLH patients and also for other HLH subgroups. Hence, all subgroups were pooled for further analysis.

**Fig. 2:**
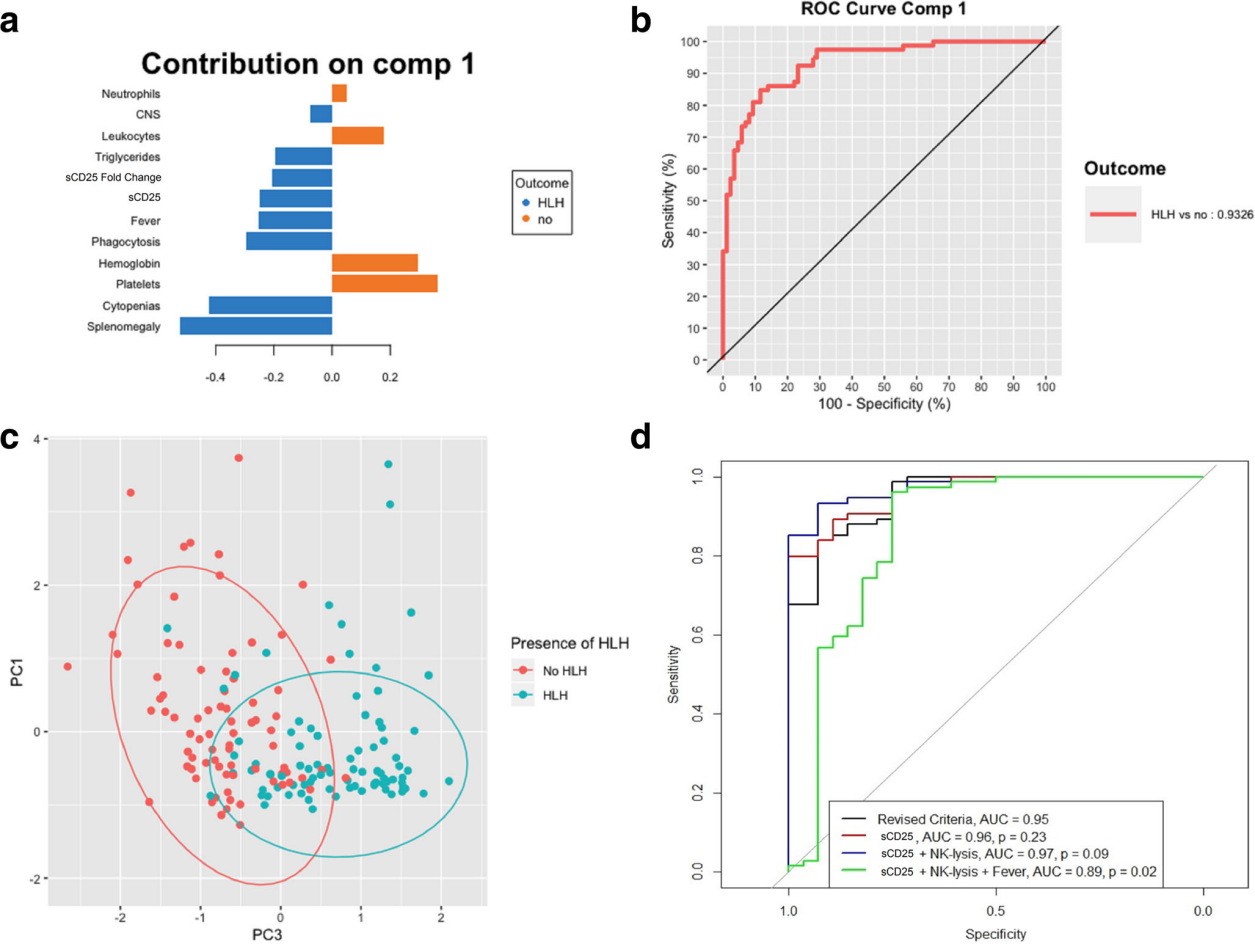
List of HLH defining symptoms that separate HLH patients from non-HLH patients in a PLS-DA (A), with an AUC of 0.93 (B). PCA showed that either PC1 (cytopenias, 32%) or PC2 (Ferritin/NK Lysis/sCD25, 25%) with PC3 (Splenomegaly/Proven biopsy of HLH, 21%) could separate HLH from non-HLH patients (C). MultiROC analysis wielded a combination of splenomegaly, proven biopsy of HLH, cytopenias, elevated ferritin, and ⇑triglycerides/⇓fibrinogen as minimal parameter set with an AUC of 0.95 which did not significantly improve with the addition of NK lysis or sCD25 (D)

The results of the pooled PLS-DA are shown in Fig. 2A and 2B. The combination of the presence of splenomegaly, together with cytopenias, proven tissue hemophagocytosis, fever, increased sCD25, and elevated triglycerides could distinguish HLH patients from non-HLH patients effectively with an area under the curve (AUC) of 0.93. Moreover, splenomegaly, biopsy-proven hemophagocytosis, and cytopenias are the most distinguishing parameters in this analysis.

### Unsupervised Clustering with Dimension Reduction

Finally, a PCA analysis with oblique rotation was performed to see if an unsupervised approach would yield similar results as the supervised approach. The Kaiser-Meyer-Olkin (KMO) measure showed that there were too few samples to explain all variables, which led to the exclusion of NK lysis 2:1, NK fold change and age, since these had the poorest common variance. This resulted in an overall KMO = 0.64 with no single value below 0.5. Bartlett’s test of sphericity resulted in chi-squared (45) = 239, *p* < 0.0001. The scree plot showed an “elbow” at four factors, indicating that four factors were sufficient to explain most variance within the dataset. This was confirmed by the dimension reduction data which showed that four factors had a cumulative variance of 67%, of which the first three explained 81% (Supplementary figure 2 & Supplementary table 3).

Cluster analysis showed that either PC1 or PC2 combined with PC3 could distinguish between the non-HLH and HLH patients (Fig. 2C). The variance in PC1 was mostly caused by fever (0.74), triglycerides (0.65) and splenomegaly (0.55), and biopsy-proven phagocytosis (0.55). For PC2, this was ferritin (0.84), NK lysis (0.96), and sCD25 (0.35) and for PC3, leukocytes (0.98), neutrophils (0.95), and platelets (0.55).

### Simulating Minimal Parameter Sets with multiROC

**Fig. 3:**
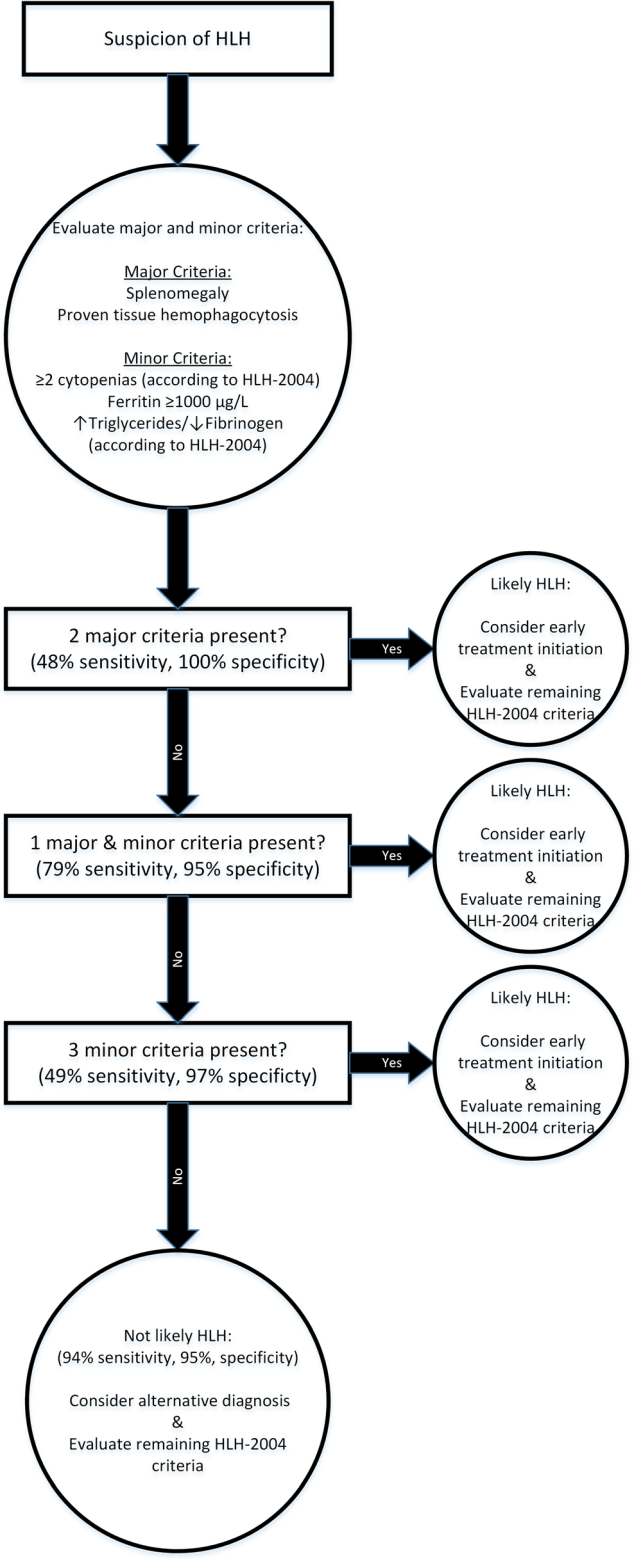
Decision tree according to minimal parameter set for diagnosing HLH and initiation of treatment. The separate sensitivity and specificity of each argument is displayed as well as the combined sensitivity and specificity of the minimal parameter set as a whole

Since tissue hemophagocytosis, splenomegaly and cytopenias were defining parameters for HLH in both the PLS-DA and the PCA; these criteria were used as initial parameter set. We then simulated the minimal parameter set needed for HLH diagnosis, by iteratively adding the criterion that caused the largest increase in AUC (Supplementary, figure 3). This ultimately led to the discovery of a combination of biopsy-proven hemophagocytosis, splenomegaly, cytopenias in ≥2 lineages, ferritin ≥1000, and ⇑triglycerides/⇓fibrinogen with an AUC of 0.95. Further addition of the other criteria (fever, sCD25, and aberrant NK/lymphocyte function assay) did not greatly improve the algorithm (Fig. 2D, AUC 0.96–097), suggesting that these parameters are not essential for the diagnosis of HLH.

Furthermore, since splenomegaly and biopsy-proven hemophagocytosis clustered together in the PCA and were also among the top discriminative parameters in the PLS-DA, these criteria were analyzed as major criteria. The remaining three criteria were analyzed as minor criteria and compared to the golden standard, the HLH-2004 criteria, as presented in Table 2. HLH was most likely when a patient either had 2 major positive criteria (48% sensitivity, 100% specificity), 1 major and 2 minor positive criteria (79% sensitivity, 95% specificity), or 3 minor positive criteria (49% sensitivity, 97% specificity), with a combined sensitivity of 94% and specificity of 95% (Table 2 and Fig. 3).

**Table 2:** Analysis of the sensitivity and specificity of the minimal parameter set that can predict HLH with splenomegaly and tissue phagocytosis as major criteria and ferritin, cytopenia, and triglycerides/fibrinogen as minor criteria. These were replicated in another retrospective cohort which produced similar results

Criteria	Test	Discovery cohort sensitivity (CI)	Discovery cohort specificity (CI)	Replication cohort sensitivity (CI)	Replication cohort specificity (CI)
Major criteria:	2 major +	0.48 (0.37–0.59)	1.0 (0.91–1.0)	0.44 (0.25–0.54)	1.0 (0.91–1.0)
- Splenomegaly					
- Proven tissue Phagocytosis	1 major and 2 minor +	0.79 (0.69–0.87)	0.95 (0.88–0.99)	0.87 (0.79–0.93)	0.97 (0.86–1.0)
Minor criteria:	3 minor +	0.49 (0.38–0.60)	0.97 (0.91–1.00)	0.76 (0.67–0.84)	0.97 (0.86–1.0)
- ≥2 cytopenias (HLH-2004)					
- Ferritin ≥1000 μg/L	Minimal parameter set	0.94 (0.86–0.98)	0.95 (0.88–0.99)	0.98 (0.94–1.0)	0.95 (0.82–0.99)
- ⇑ Triglycerides/ ⇓ Fibrinogen (HLH-2004)

### Analysis of the Minimal Parameter Set in the Replication Cohort

The replication cohort consisted of 109 sHLH patients with a median age of 58 (19–77) and 38 non-HLH patients with a median age of 54 (19–81). The most common primary diagnosis in the non-HLH group was autoimmune (47%), whereas malignancy (39.4%) and infection (36.7%) were the most common causes of sHLH in this cohort (Supplementary, table 4).

The minimal parameter set, which distinguished patients with HLH from non-HLH patients when two major criteria were positive (44% sensitivity, 100% specificity), one major and two minor criteria were positive (87% sensitivity, 97% specificity), or three minor criteria were positive (76% sensitivity, 97 specificity), could distinguish sHLH patients from non-HLH patients with 98% sensitivity and 95% specificity which confirmed the sensitivity and specificity of the minimal parameter set.

Furthermore, the presence of either splenomegaly, cytopenias in ≥2 cell lines, or ferritin ≥1000 μg/L yielded 100% sensitivity and 16% specificity.

### The Role of the NK/Lymphocyte Function and sCD25 Assays

Even though the NK/lymphocyte function and sCD25 assays are not included in the minimal parameter set, they are part of the HLH-2004 diagnostic criteria. We measured their performance in this cohort as decisive fifth criterion in borderline positive cases. There were 26 cases in which HLH was diagnosed based on the minimum of 5 positive criteria. Since sensitivity of the NK/lymphocyte function assay was low (Table 3), it could only be used as fifth positive criterion in 6/26 cases. Hence, we calculated a cutoff for the dilution series (*n* = 103, 62 non-HLH patients and 41 HLH patients) with maximum sensitivity at a specificity of at least 90%, to improve the diagnostic properties of the NK lysis assay, without impairing its robustness. This wielded a cutoff fold change of 1.17 with an AUC of 0.602, which significantly improved the diagnostic properties of the NK/lymphocyte function assay (Table 3).

**Table 3:** Analysis of the performance of the current NK lymphocyte function tests vs addition of the dilution series as sole predictor for HLH (*p < 0.01) and sensitivity and specificity cutoffs for sCD25 fold change as HLH predictor

Test	Sensitivity (95% CI)	Specificity (95% CI)
Lysis <5% and CD107a <7%	0.27 (0.07–0.31) *	0.92 (0.83–0.97)
Foldchange <1.17	0.29 (0.16–0.45)	0.94 (0.84–0.98)
Combined Criteria	0.55 (0.39–0.70) *	0.87 (0.76–0.94)
sCD25 > 2.63x upper limit	0.93 (0.84–0.98)	0.40 (0.27–0.53)
sCD25 > 11.8x upper limit	0.42 (0.31–0.55)	0.91 (0.81–0.97)

sCD25 has previously been suggested as a sensitive HLH marker. [24] In our cohort, it was needed to get to 5 positive criteria in 20/26 cases. To confirm previous findings, the performance of sCD25 as sole indicator of HLH was measured in our cohort (*n* = 120, 59 non-HLH patients, 61 HLH patients). Cutoffs were calculated with a minimum sensitivity of 90% and with a minimum specificity of 90%, which were 2.63 and 11.8 respectively with an AUC of 0.806 (Table 2). These results implicate that although NK lysis and sCD25 are not needed for initial treatment initiation, they can be used to acquire the five positive criteria needed for unambiguous diagnosis or to further support the diagnosis of HLH.

## Discussion

Early diagnosis of HLH is indispensable for timely administration of therapy to prevent aggravation of immune dysregulation, clinical deterioration, and significant morbidity and mortality. In this retrospective cohort analysis, we evaluated the diagnostic value of the individual HLH-2004 criteria using novel statistical methods including unsupervised hierarchical cluster analysis and principal component analysis. We defined a minimal parameter consisting of major and minor criteria for identification of HLH patients, which could facilitate early therapeutic decision-making for HLH.

In a rigid diagnostic approach, HLH is likely when 5/8 of the HLH-2004 criteria have been met. This may delay timely diagnosis, because several of these criteria require time-consuming assays, which are not widely available. Delayed diagnosis is especially threatening for severely ill patients. Vice versa, it has been shown that early initiation of etoposide improved survival in a retrospective study in 162 sHLH patients. Thus, any effort to facilitate an early diagnosis is important to reduce the high mortality of HLH. [12]

Failure to meet either the ferritin, cytopenia, or splenomegaly criterion excluded HLH in our cohort. At the same time, we found that this minimal parameter set consisting of 2 major criteria (hemophagocytosis and splenomegaly) and 3 minor criteria (cytopenia, increased ferritin, and increased triglycerides/low fibrinogen), which can all be available within 24 h, predicted HLH with 95% (88–99) sensitivity and 94% (86–98) specificity.

As with the HScore, which is used to facilitate early identification of HLH in suspected cases, [25, 26] the number of criteria needed to predict HLH occurrence was reduced. Fever, the NK lysis assay, and sCD25 levels were excluded from our model as initial diagnostics. An advantage of our minimal parameter set is that we used the distinction of major and minor criteria and calculated the diagnostic properties of different sets of criteria, exactly predicting the combined strength of these sets. [24] More recently, other tools have been identified such as the MAS classification criteria, the MS score, and the ESR/ferritin ratio. [27–29] These have however only been confirmed in patients with autoimmune disease-related HLH and hence, their performance in the entire spectrum of HLH is unknown. Since the minimal parameter set had a false positive rate of 1:20, we still suggest to confirm HLH in all suspected HLH patients with the full set of HLH-2004 criteria, combined with genetic confirmation and evaluation of secondary triggers where indicated, to prevent longtime exposure to immunosuppressants in non-HLH patients.

The results further showed that, in contrast to what is known from previous cohorts, individual criteria such as ferritin or sCD25 lacked either specificity or sensitivity. [15, 24, 30, 31] Patients with abnormal ferritin, sCD25, and NK lysis values formed one cluster, suggesting that these parameters are all indicators of severe systemic inflammation or critical illness. This is in line with other studies showing that NK lymphocyte–mediated target cell lysis, cytokine production, and CD107a surface receptor expression were lower in ICU patients compared to healthy controls. [32–34]

Data on the diagnostic value of ferritin as sole marker for HLH remain inconclusive. [35, 36] We now show that it can indicate patients for whom HLH diagnostics would be appropriate when assessed together with spleen size and the number and severity of cytopenias. [15, 37] We thus advocate the addition of ferritin measurement to the routine workup of patients with systemic inflammation. [35, 38–40]

Moreover, classical statistical methods and clustering analysis could not distinguish pHLH from sHLH patients in patients suspected of HLH based on the 8 HLH-2004 criteria, excluding pHLH patients in the analysis did not alter the results, showing that in our cohort at least these patients could be diagnosed identically. This was confirmed by the fact that the MHscore, which uses age of onset combined with several HLH-2004 criteria, also failed to distinguish pHLH from sHLH in this cohort. Additionally, in contrast to a previous study, patients with malignancy-induced HLH could not be distinguished from patients with HLH induced by other underlying diseases. [19, 41] This could be caused by the fact that we did not study every parameter separately, but rather the relation between the 8 criteria as a whole.

Limitations of our study warrant consideration. First, selection bias may have been introduced in this cohort, since the HLH-2004 criteria were used stringently to identify HLH patients (following current clinical practice), which may have led to underdiagnoses in this cohort, as borderline cases might have been classified as non-HLH. This could be prevented by using some of the newly proposed diagnostic tools (e.g., HScore, HLH in sJIA/SLE) and compare the outcomes with the values from the HLH-2004 criteria. This would also have enabled us to compare the performance of these scores and the newly proposed tool. Moreover, the patient selection in the discovery cohort may have been biased as only patients in whom a NK lysis assay or sCD25 was measured were included. Second, single stochastic regression imputation was used to complete missing data in the PCA dataset. This approach may have caused overidentification of interrelationships, as noise inherent to such datasets is reduced. [42] However, this effect was minimized by replicating our results in another cohort. Even though this cohort contained more adults which had other underlying etiologies, the minimal parameter set could still identify HLH patients successfully. The specificity of ferritin, cytopenias, and splenomegaly, as indicators of HLH, was lower in the replication cohort, suggesting that the non-HLH patients in this cohort were more similar to the HLH patients. This might be caused by a different approach in data inclusion or patient population. Third, although the dilution series have improved the diagnostic properties of the NK lysis assay, there was a trend towards lower specificity in comparison with the traditional NK/lymphocyte function assay. However, in the context of the 8 HLH-2004 criteria, the introduction of the dilution series did not introduce false positive cases in our cohort.

In conclusion, we determined that specific combinations of the previously existent HLH-2004 criteria provide high specificity and sensitivity for a diagnosis of HLH. The minimal HLH parameter set identified here may improve outcome of HLH patients by facilitating rapid diagnosis of HLH in patients undergoing evaluation. When confirmed in a prospective setting, this approach could be of value for timely diagnosis and treatment of HLH.

## Supplementary Information

ESM 1 (PNG 393 kb)

High Resolution (TIF 114638 kb)

ESM 2 (PNG 192 kb)

High Resolution (TIF 177575 kb)

ESM 3 (PNG 592 kb)

High Resolution (TIF 177575 kb)

ESM 4 (DOCX 8000 kb)

## Abbreviations

HLH: Hemophagocytic lymphohistiocytosis
sHLH: Secondary HLH
pHLH: Primary (familial) HLH
sCD25: Soluble cluster of differentiation 25
PLS-DA: Partial least squares differential analysis
AUC: Area under the curve
PCA: Principal component analysis
multiROC: Multi-receiver operator characteristics
