# Supplementary figures and images for: A Minimal Parameter Set Facilitating Early Decision-making in the Diagnosis of Hemophagocytic Lymphohistiocytosis
Source: J Clin Immunol. 2021 Mar 29;41(6):1219–28. doi: 10.1007/s10875-021-01005-7 (PMC8310853; doi:10.1007/s10875-021-01005-7)

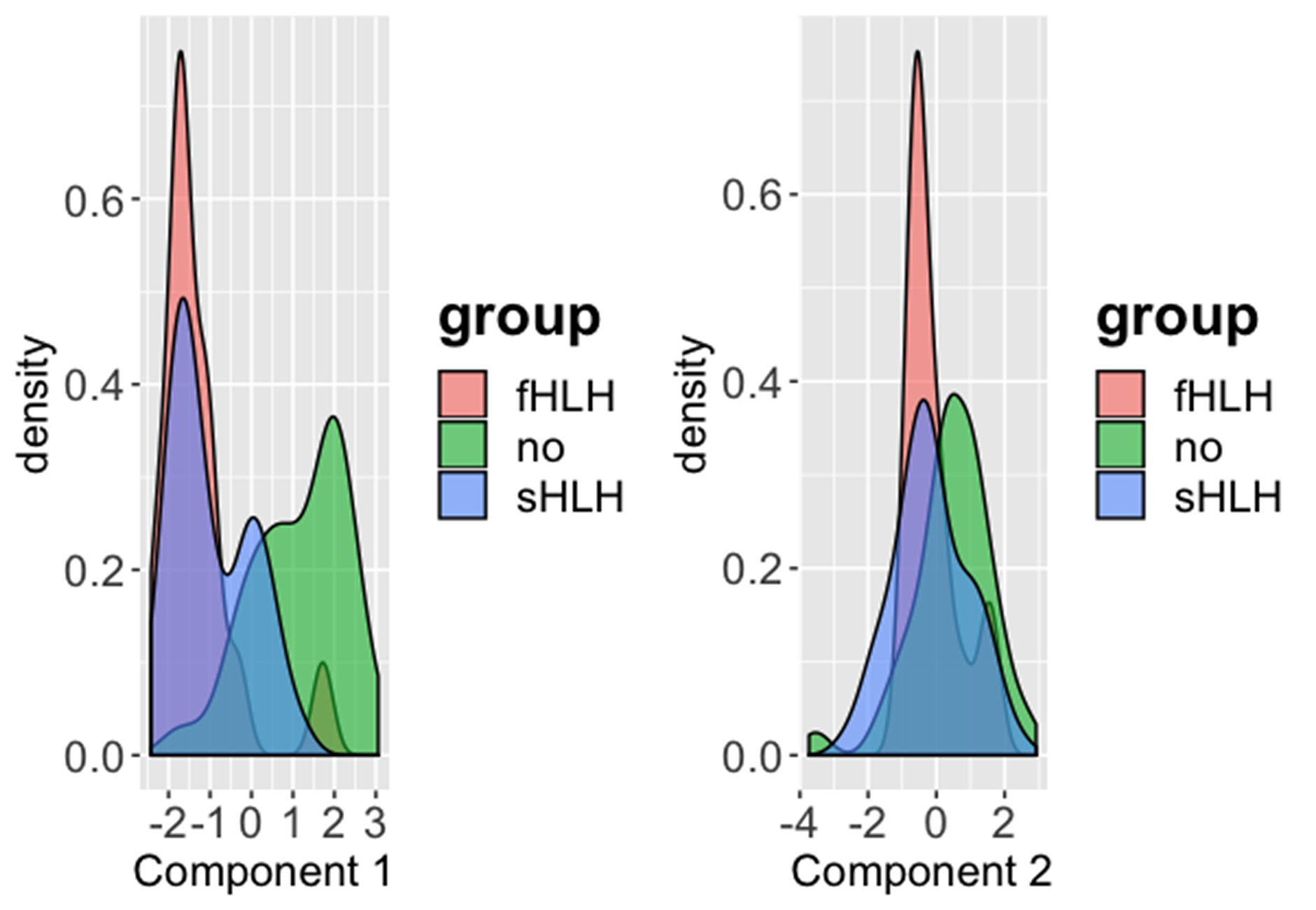

Supplement: Supplementary file 1 — (PNG 393 kb) [file 10875_2021_1005_Fig4_ESM.png]

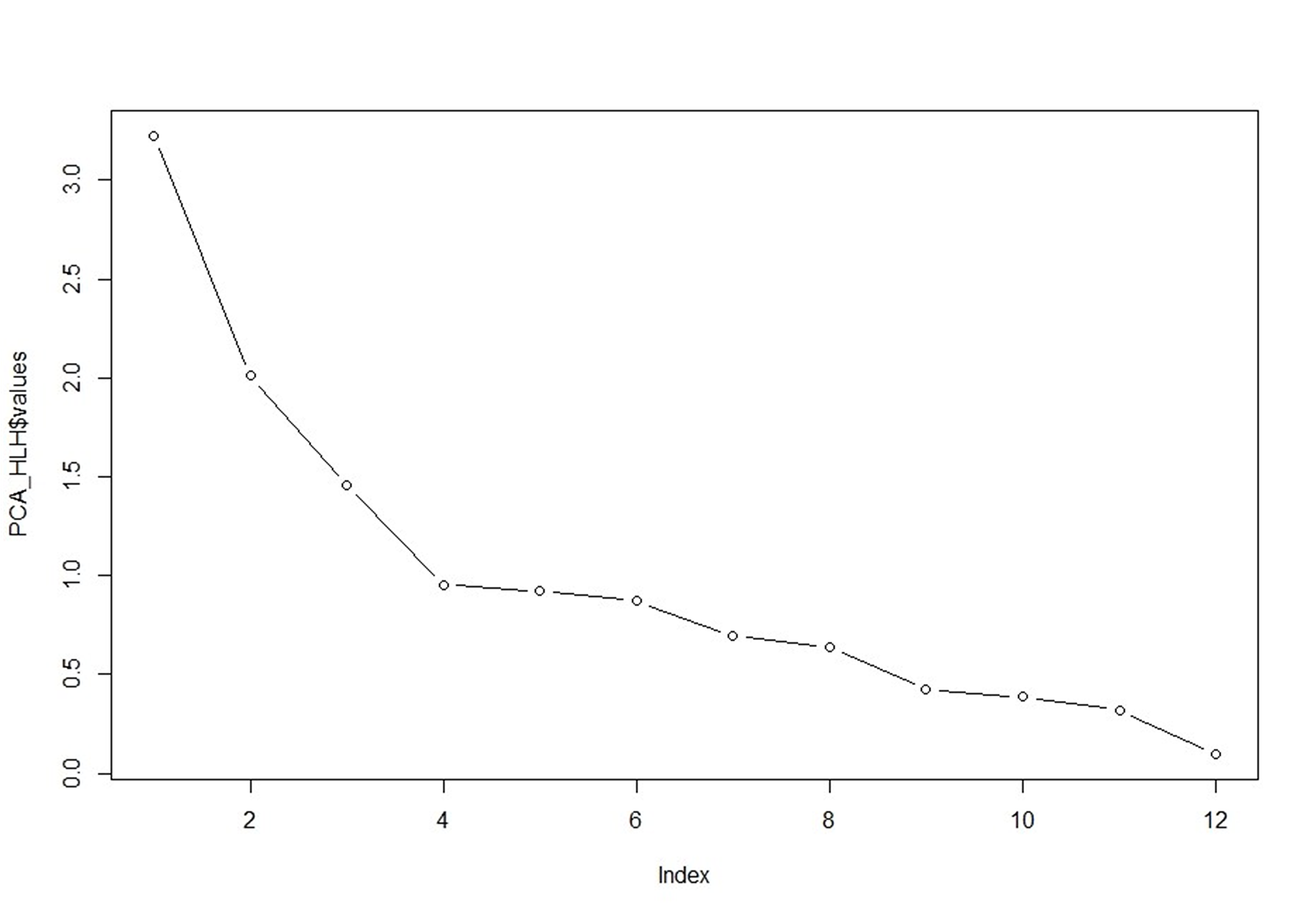

Supplement: Supplementary file 3 — (PNG 192 kb) [file 10875_2021_1005_Fig5_ESM.png]

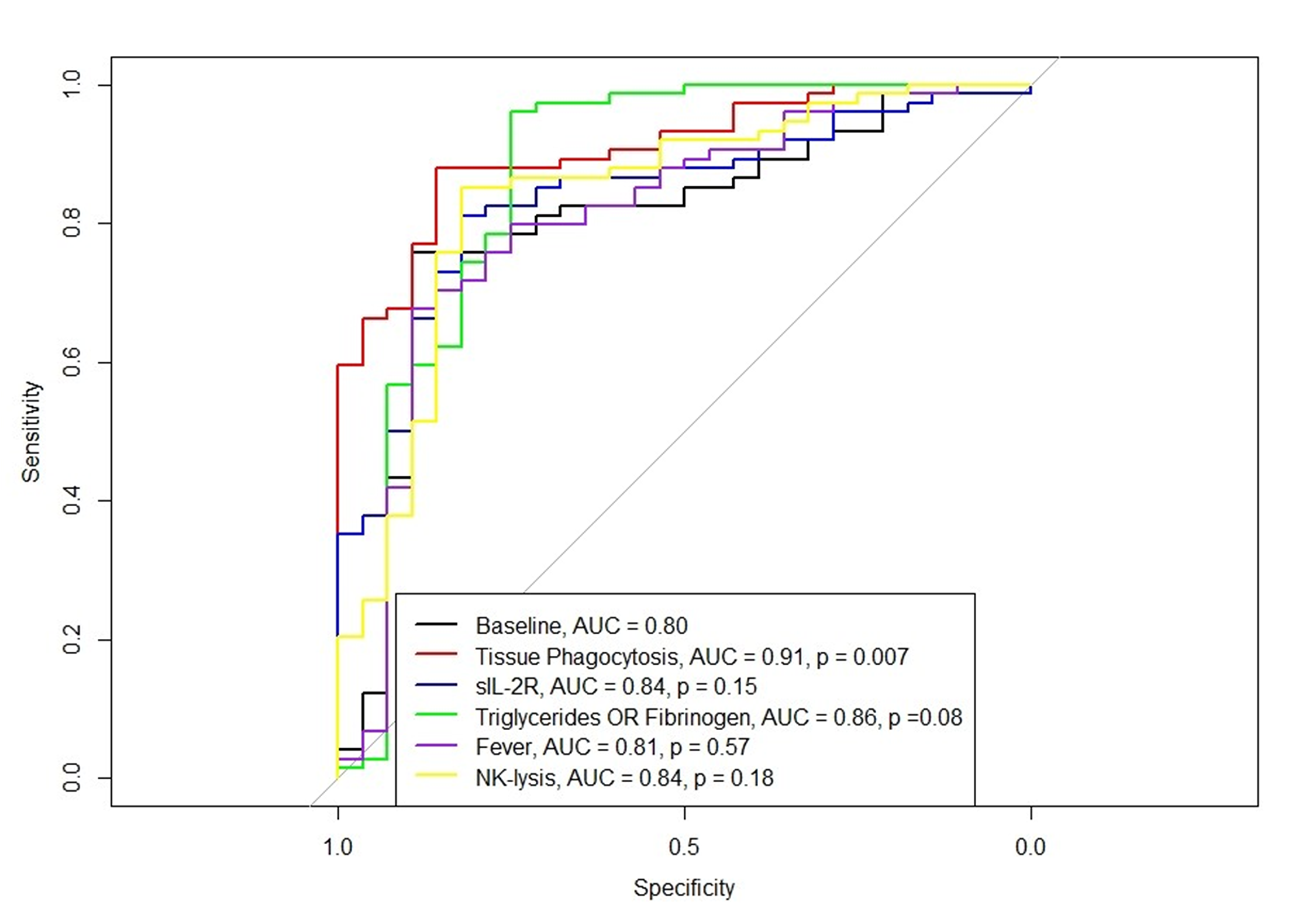

Supplement: Supplementary file 5 — (PNG 592 kb) [file 10875_2021_1005_Fig6_ESM.png]
