# Supplementary material for: A Minimal Parameter Set Facilitating Early Decision-making in the Diagnosis of Hemophagocytic Lymphohistiocytosis
Source: J Clin Immunol. 2021 Mar 29;41(6):1219–28. doi: 10.1007/s10875-021-01005-7 (PMC8310853; doi:10.1007/s10875-021-01005-7)

A minimal parameter set facilitating early decision-making in the diagnosis of hemophagocytic lymphohistiocytosis

Journal of Clinical Immunology

Bas M. Smits,^1^ Joris van Montfrans^2^, Samuel A. Merril,^3^ Lisette van de Corput,^1^ Mariëlle van Gijn,^1,4^ Andrica de Vries,^5^ Cor van den Bos,^6,7^ Floor Abbink,^7^ R.G. van der Molen,^8^ Natasja Dors,^6,9^ Caroline Lindemans,^6^ Jaap J. Boelens^1,6,10^ and Stefan Nierkens^1,6^

^1.^ Center of Translational Immunology, University Medical Center Utrecht, Utrecht, The Netherlands

^2.^ Department of Pediatric Immunology and Infectious Diseases, UMC Utrecht, The Netherlands.

^3.^ Department of Hematology, John’s Hopkins Hospital, Baltimore, United States of America

^4.^ Department of Medical Genetics, University Medical Center Utrecht, Utrecht, The Netherlands

^5.^ Department of Pediatric Oncology, Erasmus University Medical Center, Rotterdam, The Netherlands

^6.^ Princess Maxima Center for Pediatric Oncology, Utrecht, The Netherlands

^7.^ Department of Pediatric Oncology, Amsterdam University Medical Center, Amsterdam, The Netherlands

^8^Department of Laboratory Medicine, Laboratory for Medical Immunology, Radboud Institute for Molecular Life Sciences, Radboud University Medical Center

^9.^ Department of Pediatric Oncology, Radboud University Medical Center, Nijmegen, The Netherlands

^10.^ Stem Cell Transplantation and Cellular Therapies, Memorial Sloan Kettering Cancer Center, New York, NY, USA

Corresponding author:

Stefan Nierkens

Heidelberglaan 100

3584CX Utrecht

The Netherlands

| ID | Type | Gene | Homozygous | Sequence |
| --- | --- | --- | --- | --- |
| 1 | fHLH3 | UNC13D | No | c.247C>T (p.Arg83X) |
| 2 | fHLH3 | UNC13D | No | c.421G>T (p.Glu141X) en c.753+1G>T |
| 3 | fHLH2 | PRF1 | Yes | c.666C>A (p.His222Gln) |
| 4 | fHLH5 | STXBP2 | NA | NA |
| 5 | fHLH3 | UNC13D | Yes | c.2782C>T (p.Arg928Cys) |
| 6 | fHLH3 | UNC13D | NA | NA |
| 7 | RAB27A | RAB27A | NA | NA |
| 8 | fHLH5 | STXBP2 | No | c.1621G>A (p.Gly541Ser) |
| 9 | fHLH5 | STXBP2 | NA | NA |
| 10 | fHLH3 | UNC13D | Yes | c.1055 +1 G>A |
| 11 | fHLH2 | PRF1 | Yes | NA |
| 12 | fHLH2 | PRF1 | No | compound heterozygous: c.445G>A (p.Gly149Ser),  c.757G>A (p.Glu253Lys) |
| 13 | fHLH4 | STX11 | NA | NA |
| 14 | fHLH5 | STXBP2 | No | compound heterozygous: c.1247-1G>C, p.(?),  c.1621G>A, p.(Gly541Ser) |
| 15 | NA | NA | NA | NA |
| 16 | fHLH4 | STX11 | NA | NA |

Supplementary Table 1: pHLH type, affected gene and sequence of the pHLH patients included in this study

| Test | Primary Sensitivity (CI) | Primary Specificity (CI) | Replicated Sensitivity (CI) | Replicated Specificity (CI) |
| --- | --- | --- | --- | --- |
| 1/3 Positive criteria | 1.0 (0.96 – 1.0) | 0.65 (0.56 – 0.74) | 1.0 (0.97 – 1.0) | 0.16 (0.06 – 0.31) |
|  |  |  |  |  |
| 2/3 Positive criteria | 0.77 (0.67 – 0.86) | 0.92 (0.85 – 0.96) | 0.98 (0.94 – 1.0) | 0.45 (0.29 – 0.62) |

Supplementary Table 2: The performance of splenomegaly, ferritin and cytopenia as screening criteria for HLH. Predictive performance was calculated for 1/3 positive criteria and 2/3 positive criteria

|  | PC1 | PC2 | TC3 | TC4 | h2 | u2 | com |
| --- | --- | --- | --- | --- | --- | --- | --- |
| NK lysis ER 1:1 |  | 0.96 |  |  | 0.94 | 0.061 | 1 |
| NK lysis ER 2:1 |  | 0.95 |  |  | 0.93 | 0.066 | 1 |
| Ferritine |  | 0.84 |  |  | 0.71 | 0.289 | 1.1 |
| Hemoglobine | -0.74 |  |  |  | 0.52 | 0.48 | 1.2 |
| Fever | 0.74 |  |  |  | 0.51 | 0.485 | 1.1 |
| Triglyceride | 0.65 |  |  |  | 0.51 | 0.488 | 1.2 |
| Biopsy proven phagocytosis | 0.55 |  |  |  | 0.43 | 0.571 | 1.4 |
| Splenomegaly | 0.55 |  |  |  | 0.45 | 0.547 | 1.5 |
| sCD25 | 0.4 | 0.35 |  | -0.4 | 0.52 | 0.477 | 3 |
| Leukocytes |  |  | 0.98 |  | 0.92 | 0.085 | 1 |
| Neutrophils |  |  | 0.95 |  | 0.9 | 0.1 | 1 |
| Platelets |  |  | 0.55 |  | 0.57 | 0.43 | 1.9 |
| Fibrinogen |  |  |  | 0.9 | 0.79 | 0.206 | 1 |
|  |  |  |  |  |  |  |  |
| SS Loadings | 2.44 | 2.72 | 2.226 | 1.29 |  |  |  |
| Proportion of Variance | 0.21 | 0.19 | 0.17 | 0.1 |  |  |  |
| Cumulative Variance | 0.21 | 0.4 | 0.57 | 0.67 |  |  |  |
| Proportion Explained | 0.31 | 0.28 | 0.26 | 0.15 |  |  |  |
| Cumulative Proportion | 0.31 | 0.59 | 0.85 | 1 |  |  |  |

Supplementary Table 2: Raw data of the Principal Component analysis showing the different factor loadings and the corresponding variance and proportion. PC1 was mostly explained by fever, triglyceride value, splenomegaly and biopsy proven phagocytosis. PC2 was mostly explained by NK lysis, Ferritine and SIL-2Ralpha levels and PC3 was mostly explained by leukocytes, neutrophils & platelets. The cumulative proportion of these 3 components was 85% explaining 57% of the variance within the dataset.

|  | non-HLH | sHLH |
| --- | --- | --- |
| Number of Patients (n) | 18 | 109 |
| Median age (years) | 54 (19 – 81) | 58 (19 – 77) |
| Gender (% Male) | 52.6 | 59.6 |
| Type of Disease   - Auto-Immune - Immunodeficiency - Genetic (non-HLH) - Genetic (HLH) - Infection - Malignancy - Unknown - Other | 18  0  1  0  10  5  1  3 | 15  0  1  0  40  43  9  0 |
| Overall Survival (%) | 71.1 | 38.5 |

Supplementary Table 3: Summary statistics on the patients in the replication cohort

**Supplementary figure 1:** Overlap of the HLH specific diagnostic criteria found by PLS-DA between fHLH, sHLH and non-HLH patients for both component 1 (left) and component 2 (right), showing an overlap between fHLH and sHLH patients.


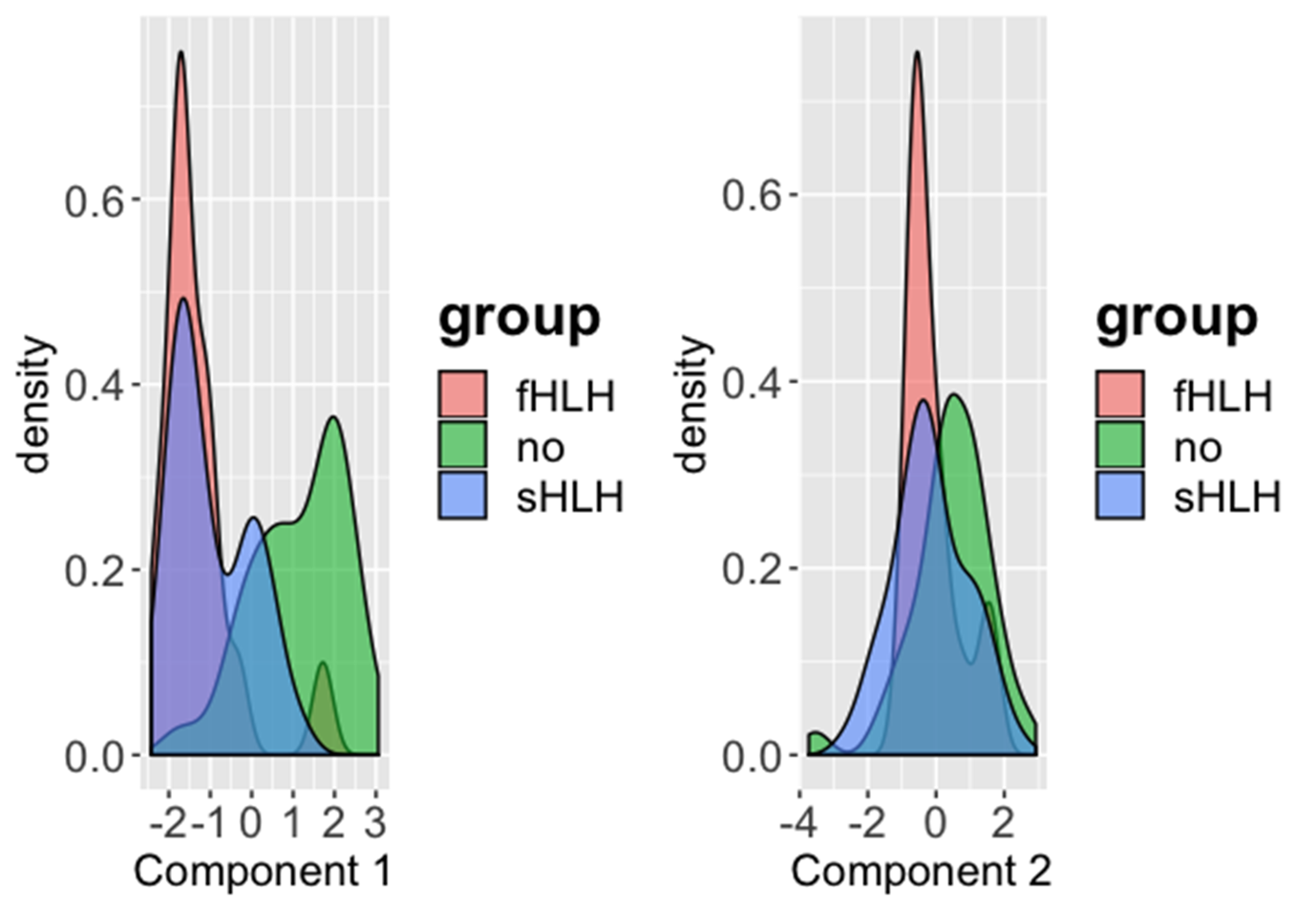


**Supplementary figure 2:** Scree plot of the PCA analysis showing an “elbow” shape at 4 components, indicating that 4 components are sufficient to explain most variance that occurs within the data.


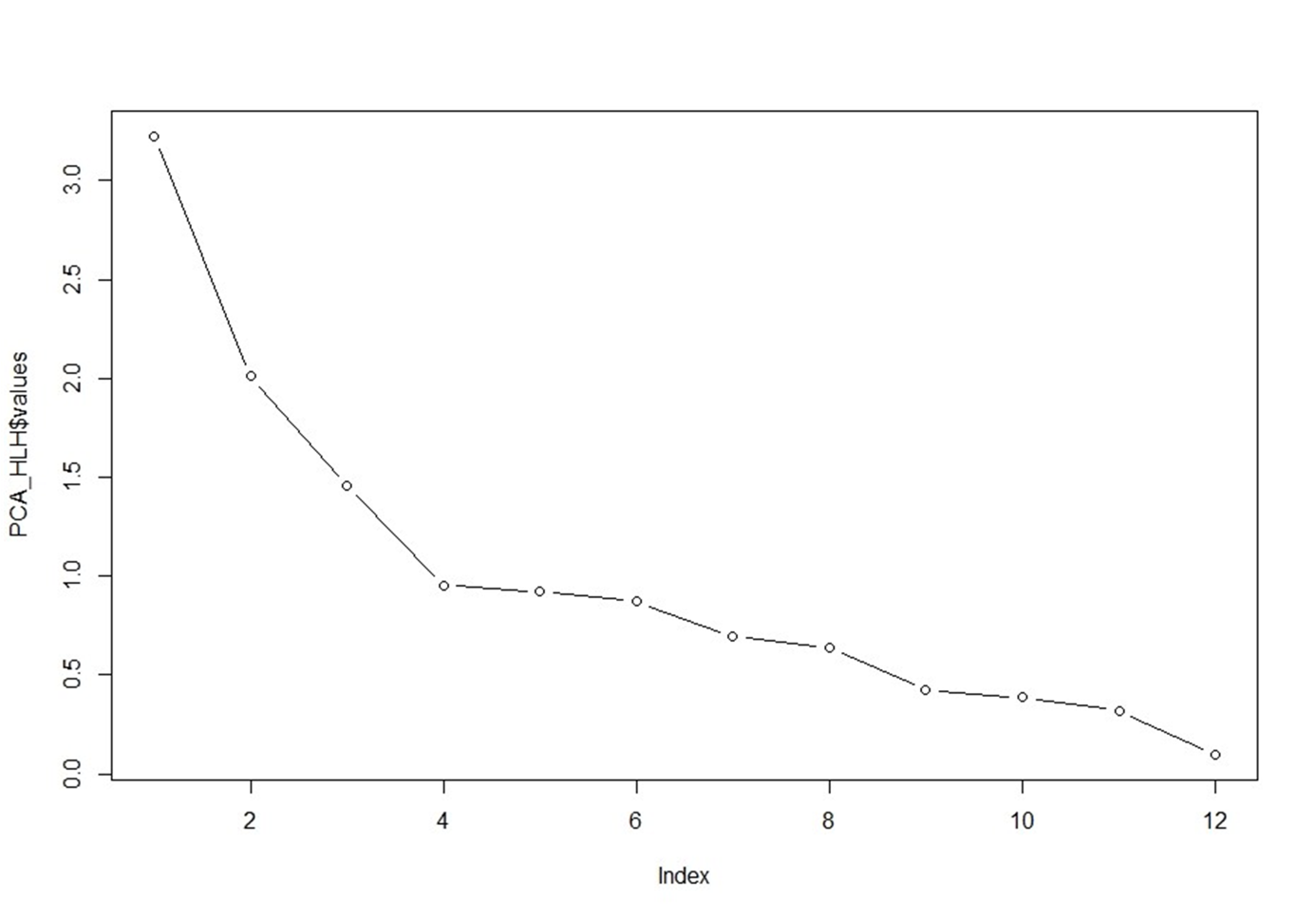


**Supplementary figure 3:** multiROC that shows the increase in AUC with addition of the separate HLH-2004 criteria to the baseline model.


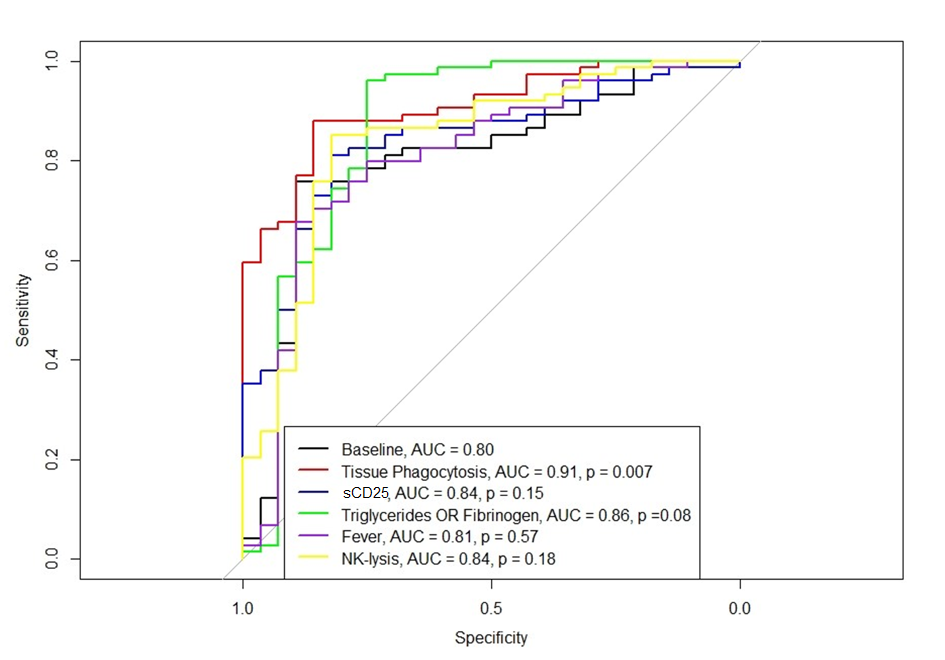

Supplement: Supplementary file 7 — (DOCX 8000 kb) [file 10875_2021_1005_MOESM4_ESM.docx]
